# The ankyrin repeat gene family in *Capsicum* spp: Genome-wide survey, characterization and gene expression profile

**DOI:** 10.1038/s41598-020-61057-4

**Published:** 2020-03-04

**Authors:** Carlos Lopez-Ortiz, Yadira Peña-Garcia, Purushothaman Natarajan, Menuka Bhandari, Venkata Abburi, Sudip Kumar Dutta, Lav Yadav, John Stommel, Padma Nimmakayala, Umesh K. Reddy

**Affiliations:** 1 Department of Biology, Gus R. Douglass Institute, West Virginia State University, Institute, West Virginia, United States of America; 2 Department of Genetic Engineering, School of Bioengineering, SRM Institute of Science and Technology, Kattankulathur, 603203 India; 3 ICAR RC NEH Region, Mizoram Centre, Kolasib, Mizoram India; 4 Genetic Improvement of Fruits and Vegetables Laboratory (USDA, ARS), Beltsville, MD 20705 USA

**Keywords:** Natural variation in plants, Plant genetics

## Abstract

The ankyrin (ANK) repeat protein family is largely distributed across plants and has been found to participate in multiple processes such as plant growth and development, hormone response, response to biotic and abiotic stresses. It is considered as one of the major markers of capsaicin content in pepper fruits. In this study, we performed a genome-wide identification and expression analysis of genes encoding ANK proteins in three *Capsicum* species: *Capsicum baccatum*, *Capsicum annuum* and *Capsicum chinense*. We identified a total of 87, 85 and 96 *ANK* genes in *C. baccatum*, *C. annuum* and *C. chinense* genomes, respectively. Next, we performed a comprehensive bioinformatics analysis of the *Capsicum ANK* gene family including gene chromosomal localization, *Cis*-elements, conserved motif identification, intron/exon structural patterns and gene ontology classification as well as profile expression. Phylogenetic and domain organization analysis grouped the *Capsicum ANK* gene family into ten subfamilies distributed across all 12 pepper chromosomes at different densities. Analysis of the expression of *ANK* genes in leaf and pepper fruits suggested that the *ANKs* have specific expression patterns at various developmental stages in placenta tissue. Our results provide valuable information for further studies of the evolution, classification and putative functions of *ANK* genes in pepper.

## Introduction

The ankyrin (ANK) repeat domain is one of the most common conserved protein domains widely distributed in different organisms ranging from viruses to humans^1^. This protein domain was identified for the first time in the yeast cell-cycle regulators Swi6 and Cdc10^2^ and in the *Drosophila melanogaster* signaling protein Notch^3^. The name of these proteins was assigned after the cytoskeletal ankyrin protein was found to contain 24 copies of this sequence^4^. ANK repeats contain a ~33-residue motif repeated in tandem and consisting of two antiparallel α-helices connected by a series of inverting β-hairpin motifs. Indeed, the ANK domain is better characterized by a folding structure rather than functional requirements^5,6^. Although the ANK domain proteins are not known to have a specific function, they play important roles in several biological activities and have been identified in abundant different proteins with diverse functions^7^. For instance, these repeats can mediate protein–protein interactions^8,9^ and may serve as molecular chaperones^10^.

ANK proteins reported in plants are classified into 13 subfamilies based on different domains that have been identified by genome structure studies and gene expression profiles^6^. ANK domain-containing proteins of plants are usually involved in crucial physiological and developmental processes such as signaling and growth^11^, plastid differentiation^12^, embryogenesis^13^, chloroplast biogenesis^14^, formation of grana^15^, leaf morphogenesis^16^, pollen germination and polarized pollen tube growth^17,18^. Additionally, the ANK repeat-containing proteins play important roles in the response to both biotic and abiotic stresses. These proteins have been observed to participate in drought tolerance^19^, ABA-mediated regulation of reactive oxygen species levels under salt-stress^20^, and several plant diseases^21^, including those generated by fungus such as rice blast^22^.

The release of genomic data and the development of bioinformatics analyses have led to comprehensive research on the identification and characterization of the *ANK* gene family in plants such as *Arabidopsis*^23^, rice^18^, tomato^24^, maize^25^, *Physcomitrella patens*^26^ and soybean^27^. The number of ANK repeats, genes, and proteins in plants varies considerably across diverse plant species. In *Arabidopsis thaliana*, 509 ANK repeats encoded by 105 genes were reported, whereas rice contains 175 ANK repeat genes^18,23^.

Pepper (*Capsicum* spp.) is a member of the Solanaceae family and is closely related to potato, tomato, eggplant, tobacco and petunia. Pepper represents an economically important horticultural crop worldwide because of its wide variety of uses, as a food, coloring agent, and spice and in pharmaceuticals, cosmetics and ornamental products as well as for its nutrimental value^28,29^. Despite the importance of pepper, genome-wide studies remain limited. The recent whole-genome sequencing of pepper^28^ provided an excellent tool for genome-wide analysis for the identification and characterization of entire gene families present in this crop^30–32^. Recently, the ANK repeat domain was identified as one of the major markers linked to capsaicinoid synthesis in *Capsicum annuum*^33^ and *Capsicum chinense*^34^.

The present study aimed to analyze the gene locus and chromosome localization, protein length, number of ANK repeats, molecular weight (MW), isoelectric points (pI), gene structure and phylogenetic relationship of *ANK* genes in three *Capsicum* species: *Capsicum baccatum*, *C. annuum* and *C. chinense*. We also surveyed the expression patterns of *ANK* genes of *C. annuum* and *C. chinense*. These analyses will contribute to a better understanding of the evolution, function and future insights for research of the *ANK* gene family in *Capsicum* species and will also provide a robust database for the *Capsicum* research community.

## Results

### Genome-wide identification of ANK proteins in pepper

We used the conserved amino acid sequence of the ankyrin domain (Accession no. PF00023) to search the three pepper genomes in the PGP, with the HMM profile used as a query and identified 268 genes potentially encoding ANK proteins across the three genome databases: 87 in *C. baccatum* (*CbANK*), 85 in *C. annuum* (*CaANK*) and 96 in *C*. *chinense* (*CcANK*). Each of these ANK protein sequences were verified by SMART and Pfam analyses. For convenience, in this study we provide a simplified nomenclature for each identified *ANK* gene. We designated the acronyms *CbANK1* to *CbANK87* for *C. baccatum*, *CaANK1* to *CaANK85* for *C. annuum* and *CcANK1* to *CcANK96* for *C. chinense*, based on the order of appearance on chromosomes 1 to 12. Length, MW and pI of ANK proteins were deduced from their protein sequences and are in Tables S1–S3. The protein length of *CbANKs* ranged from 102 to 842 residues, *CaANKs* from 117 to 958 residues, and *CcANKs* from 81 to 760 residues. Hence, we identified 1541 ANK domains within these 268 proteins across all three *Capsicum* species. The number of ANK repeats varied greatly, from 1 to 19 repeats per protein; however, the proteins with 2 to 5 ANK repeats were the most common among all the species (Fig. S1).

**Figure 1:**
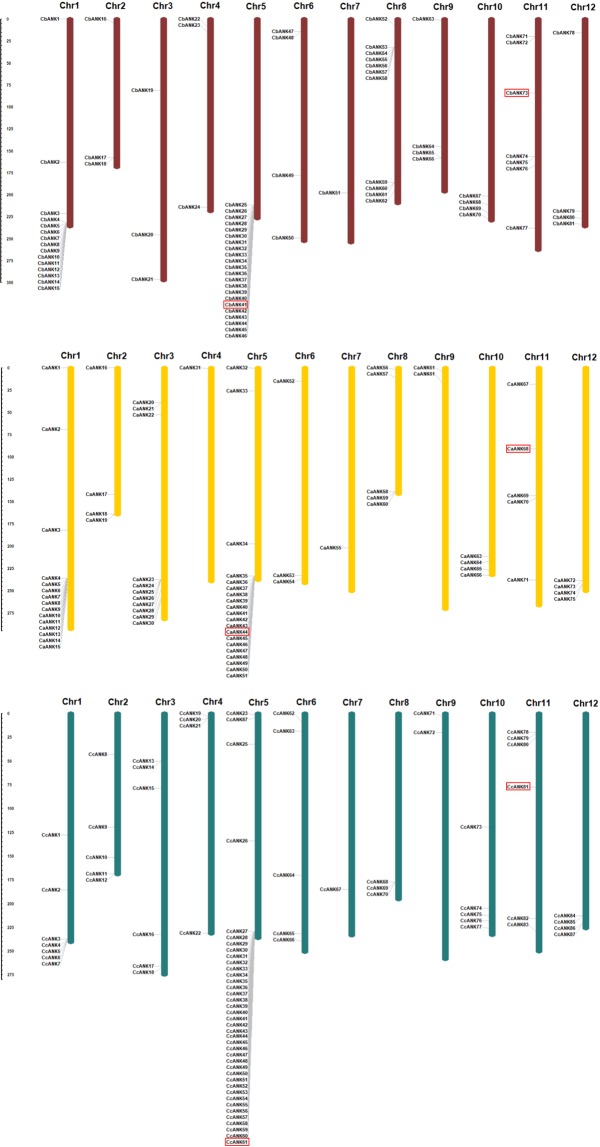
Chromosomal locations of ankyrin (ANK) proteins in the pepper *Capsicum baccatum* (red), *C. annuum* (yellow) and *C. chinense* (green). Chromosome numbers are represented at the top of each chromosome. The left panel scale indicates the chromosome length in Mbp. Orthologs genes of CA05g18080 and CA11g09160 are represented by red boxes.

Among the 268 *ANK* genes identified, 243 (92.4%) were physically mapped and unevenly distributed across the 12 chromosomes of pepper at different densities, whereas the other 25 genes were located on scaffolds (Fig. 1). Among all chromosomes and species, chromosome 5 of *C. chinense* had the highest number of *ANK* genes, 39 (~41%), followed by *C. baccatum*, 22 (25.2%), and *C. annuum*, 20 (23.52%). Chromosome 1 of *C. baccatum* and *C. annuum* had 15 *ANK* genes, and chromosomes 8 and 1 of *C. baccatum* and *C. annuum* had 11 *ANK* genes. Among all species, chromosome 7 contained the lowest number of *ANK* genes, only one.

The distribution pattern of *ANK* genes on individual chromosomes also indicated physical regions with a relatively higher accumulation of multiple *ANK* gene clusters, such as chromosome 5 at the lower end of the arm for all species, with higher density in *C. chinense*. The distribution and density of *ANK* gene clusters differed among the three genomes. For example, chromosome 1 of *C. baccatum* and *C. annuum* had a high-density cluster, which was not in the same density in *C. chinense*. Another cluster was found in the lower arm of chromosome 3 but only in *C. annuum* and just few genes were found in *C. baccatum* and *C. chinense*. In chromosome 8, *ANK* genes were distributed in the upper part of *C. baccatum* and *C. annuum* but not *C. chinense*. Similarly, one *ANK* gene was present in the upper end of chromosome 12 for C*. baccatum* but was absent in the other two pepper species.

### Identification of conserved motifs of *ANK* genes in *Capsicum* and domain analysis

To analyze the function and domain distribution of the putative *Capsicum ANK* genes, Pfam and Hmmer platforms were used for protein analysis, to allow for identifying the major domains. As a result, the 268 *ANK* genes were classified into 10 subfamilies based on their domain composition: ANK-U, ANK-TM, ANK-PK, ANK-ZnF, ANK-BTB, ANK-BPA, ANK-ACBP, ANK-GPCR, ANK-IQ and ANK-O (Table 1). Among these genes, 42 ANK domain-containing proteins were found in *C. baccatum* and 45 in *C. annuum*, whereas in *C. chinense*, 62 proteins with a unique ANK domain (ANK-U subfamily) were reported. The transmembrane domain (ANK-TM) was the second most abundant subfamily, containing 33, 29, and 26 proteins in *C. baccatum, C. annuum*, and *C. chinense*, respectively. The ANK-ZnF (zinc-finger) subfamily contained two members in each species, which were classified into the subgroup ANK-CCCH based on the type of zinc finger. In the same way, *C. annuum* and *C. baccatum* featured the tramtrack and bricabrac domains, two members of the ANK-BTB-containing broad complex, but only one (*CcANK23*) was identified in *C. chinense*. One gene belonging to the ANK-ACBP (Acyl-CoA-binding protein) subfamily and one more from the ANK-GPCR subfamily, which contains a GPCR-chapero-1, were found in all *Capsicum* species analyzed. *C. baccatum* and *C. chinense* but not *C. annuum* had a member of the protein kinase domain family, ANK-PK, containing serine/threonine or tyrosine kinase. *C. annuum* and *C. baccatum* shared one gene in common that belongs to the ANK-BPA family, containing the BAR, PH and ArfGap domains. Only *C. annuum* featured the ANK-IQ subfamily, containing the calmodulin-binding domain with only one member. The remaining proteins were grouped into the ANK-O subfamily, containing different domains including the motile-sperm, bromodomain, STI1, UreD, G-patch, bVFLR1 and Myb DNA binding domains.

**Table 1:** Comparative analysis of ankyrin (ANK) repeat proteins between *Capsicum* and other plant species.

Specie	ANK Subfamily												# of ANK proteins	# of Proteins	% of ANK proteins	Reference
	U	TM	ZnF	BTB	ACBP	GPCR	PK	BPA	IQ	RF	TPR	O				
*A. thaliana*	18	40	6	7	2	0	7	4	4	5	1	11	105	25,498	0.41	^23^
*O. sativa*	73	37	7	6	0	0	4	3	4	9	22	10	175	35,825	0.49	^18^
*Z. mays*	30	15	3	2	0	0	4	2	1	9	2	3	71	39,591	0.18	^25^
*P. patens*	21	0	3	3	3	2	9	3	0	6	0	4	54	35,398	0.15	^26^
*E. siliculosus*	178	0	7	0	0	0	56	3	8	3	2	82	339	16,256	2.08	^38^
*G. max*	48	30	13	3	2	4	12	7	13	2	1	27	162	55,897	0.29	^27^
*S. lycopersicum*	26	25	8	7	0	4	9	4	7	7	4	26	130	33,952	0.38	^24^
*C. baccatum*	42	33	2	2	1	1	1	1	0	0	0	4	87	35,874	0.24	
*C. annuum*	45	29	2	2	1	1	0	1	1	0	0	3	85	35,884	0.24	
*C. chinense*	62	26	2	1	1	1	1	0	0	0	0	2	96	35,009	0.27	

U ankyrin repeat; TM transmembrane; ZnF, zinc finger; BTB, Broad-Complex, Tramtrack and Bric a brac; ACBP, Acyl CoA binding protein domain; GPCR, G protein-coupled receptors; PK, protein kinase domain; BPA, BAR domain, Pleckstrin homology domain and ArfGTPase- activating domain; IQ, Short calmodulin-binding motif containing conserved Ile and Gln residues; RF, RING finger; TPR, tetratricopeptide repeats; O, others domains.

The conserved motifs of ANK proteins were analyzed with the MEME server. The number of conserved motifs in each *Capsicum* ANK protein ranged from 2 to 9 (Fig. S2). Moreover, the length of the motifs ranged from 15 to 50 amino acids (Fig. S3, Table S4). Motif 5 was conserved in the ANK-U subfamily among the three *Capsicum* species, whereas motifs 8, 9 and 10 were identified in ANK-O and ANK-U subfamilies. Additionally, motifs 1, 2 and 3 were distinctively detected in all *Capsicum ANK* genes forming the configurations of ANK domain. Motifs 4 and 7 were evenly distributed in *C. annuum* and motif 6 in *C. baccatum*. The information obtained from ScanProsite analysis revealed that the function of most of the motifs was related to not only ANK domain-containing proteins but also ACCELERATED CELL DEATH 6-like protein (*ACD6*).

### Phylogenetic tree and gene structure of *Capsicum ANK* genes

**Figure 2:**
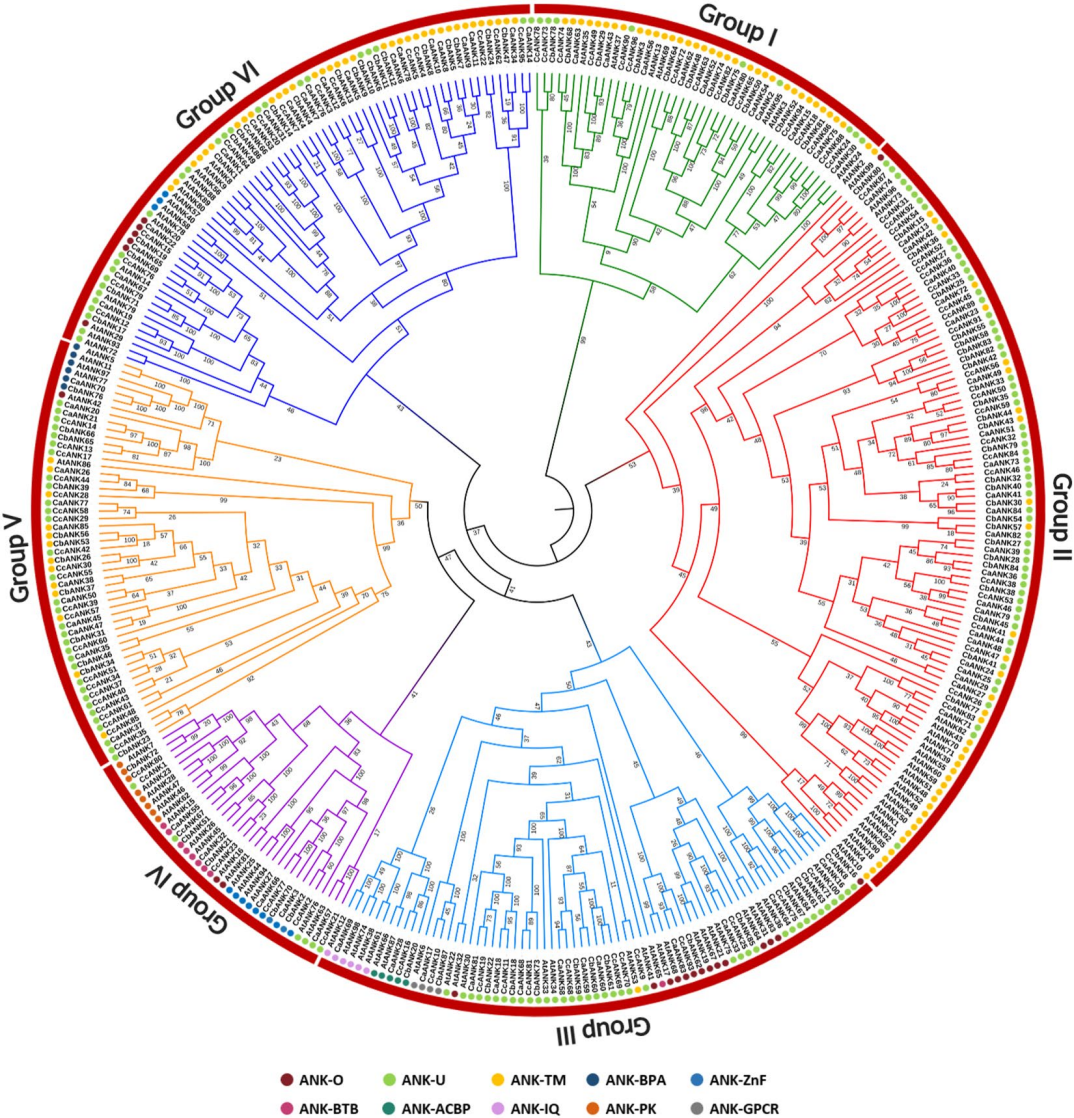
Phylogenetic relationships of *Capsicum* species and *Arabidopsis* ANK proteins. Phylogenetic analysis by the maximum-likelihood method with 1000 bootstrap replicates of the 268 and 105 ANK proteins identified from *Capsicum* species and *Arabidopsis*, respectively. ANK subfamilies are represented by different colored dots.

To gain insight into the evolution of *ANK* genes and infer their function based on homologs present in other plant species, the protein sequences of *Capsicum* species and 105 full-length ANK protein sequences from *Arabidopsis* were aligned by using MEGAX. We expanded the maximum-likelihood method with 1000 bootstrap replication to construct an unrooted phylogenetic tree (Fig. 2). In total, 373 ANK proteins from the four species were clearly divided into six major groups (groups I to VI). Most of the members in the same groups shared one or more domains outside of the ANK domain, which further supported the subfamily definition described above. For example, the ANK-U subfamily was distributed throughout all the groups; similarly, the ANK-TM subfamily was present in most of the groups except for groups III and IV. The ANK-IQ, ANK-GPCR and ANK-ACBP subfamilies were placed in group III, and the subfamilies ANK-ZnF, ANK-PK and ANK-BTB were in group IV. Finally, the ANK-BPA subfamily was placed in group V.

**Figure 3:**
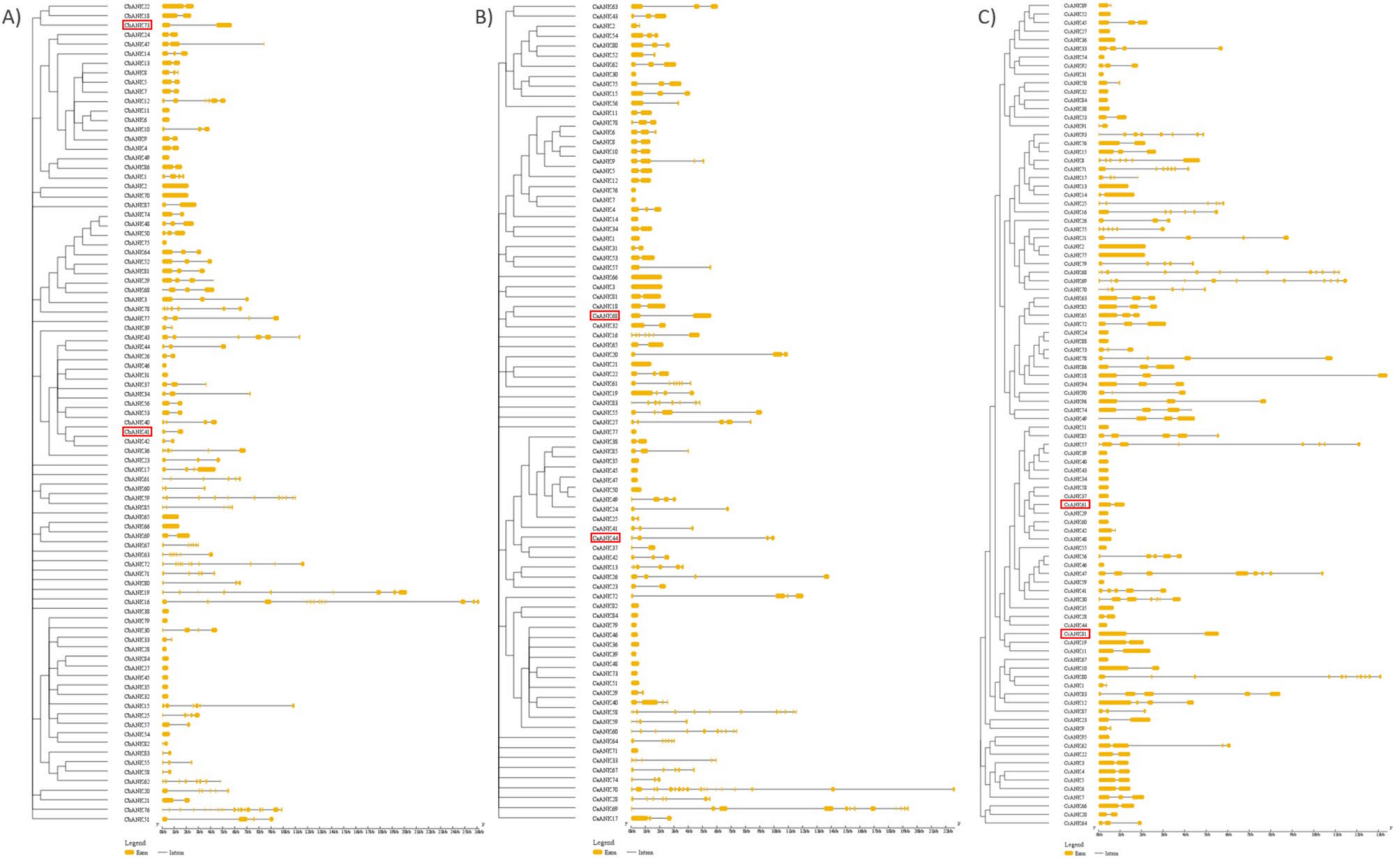
Gene structure analysis of the ANK proteins in *Capsicum* species: (**A**) *C. baccatum*, (**B**) *C. annuum* and (**C**) *C. chinense*. The exons and introns are represented by the yellow boxes and black lines, respectively. The scale bar in the bottom represents the gene length in kb. Orthologs genes of CA05g18080 and CA11g09160 are represented by red boxes.

Next, we compared the structural diversity between *Capsicum ANK* genes in terms of exon/intron arrangement of the coding and genome sequences by using the GSDS tool for generating gene structure schematic diagrams. Structure analyses of the *ANK* genes revealed that the positions, length and number of introns varied across all species and subfamilies. The number of introns present ranged from 0 to 18 in *C. baccatum* and *C*. *annuum* genes and from 0 to 11 in *C. chinense* genes. Detailed gene structure of the *Capsicum ANK* genes is in Fig. 3. *CaANK44* showed three introns, with only one in *CbANK41* and *CcANK61*. In total, 19, 23 and 30 genes contained only one exon in *C. baccatum, C. annuum* and *C. chinense*, respectively. *C. baccatum* and *C. annuum* shared one gene with 19 exons, and the maximum number of exons in *C. chinense*, 12, was found in *CcANK68*. The most closely related *ANK* genes in the same subfamily shared a similar gene structure in terms of intron number and intron-exon length.

We determined the protein structures of CA05g18080 orthologs (*CbANK41*, *CaANK44*, *CcANK61*) for each *Capsicum* species. The protein structure of *Capsicum* ANK proteins was modelled at >90% confidence using the alignment of hidden Markov models via an HMM-HMM search at the Phyre2 server^35^. Overall, ANKs appear to have very similar structures, with an α-solenoid fold architecture and secondary structure predominantly consisted of α-helices (Fig. S4) according to the classification defined by Kajava (2012)^36^. Hence, all predicted protein structures are considered highly reliable, which offers a preliminary basis for understanding the molecular function of *Capsicum* ANK proteins.

### Prediction of *Capsicum ANK* gene promoter elements

To identify putative *cis*-elements in *Capsicum* ANK promoters, we analyzed 1500-bp DNA sequences upstream of the start codon (ATG) at the Plant *Cis*-acting Regulatory DNA Elements (PLACE) website. The analysis identified 124 common *cis*-elements among all *Capsicum ANK* genes (Table S4). Furthermore, we revealed four common cis-regulatory elements — WBOXATNPR1, ASF1MOTIFCAMV, GCCCORE and SEBFCONSSTPR10A — which are involved in response to plant hormones, including auxin and salicylic acid (SA), as well as disease resistance. These results agree with the known role of *ANK* genes in plant resistance to biotic and abiotic stresses. Among the 124 *Cis-*elements, 15 were related to auxin, ABA and SA, and another six to gibberellic acid (GA). We also identified IBOX, -10PEHVPSBD, TBOXATGAPB, INRNTPSADB and GT1CONSENSUS, which have been found required for transcriptional regulation by light. Other *Cis*-elements present, MYBCORE, MYCATERD1, MYCATRD22, MYBATRD22 and MYB2AT, are related to water stress and dehydration. Finally, 11 were associated with binding protein site function. Overall, most of the predicted C*is-*elements play a role in the response to signaling hormones and are involved in different stresses.

### Syntenic *Capsicum ANK* paralog pairs

To examine the impact of duplications on the *ANK* gene family, we analyzed tandem and segmental duplication events. Syntenic paralog pairs were identified between and within the three *Capsicum* genomes and the syntenic relationships of *ANK* genes among *Capsicum* species and *Arabidopsis* genomes were visualized by generating a circular plot (Fig. S5). We found 23 pairs of ANK syntenic paralogs across all species (Table 2); 16 were intra-species and the remaining were inter-species. Among the seven inter-species pairs, four segmental duplications were intra-chromosomal, located on chromosomes 5 and 1. Most of the duplicated paralog pairs belong to the same subfamily, with exception of the segmental duplication *CbANK72*-*CcANK1*. The paralogs of this last pair were identified in chromosomes 11 and 1 and belong to the ANK-PK and ANK-U subfamilies, respectively. The last two duplication pairs were found in scaffold positions.

**Table 2:** Ka-Ks calculation for each pair of syntenic Capsicum ANK paralogs.

Syntenic paralog pairs	S-Sites	N-Sites	Ka	Ks	Ka/Ks	Selection pressure	Duplication time (MYA)
*CbANK*3-*CcANK*96	371.91	1197.09	0.00	0.02	0.15	Purifying selection	0.11
*CaANK*25-*CaANK*24	96.22	320.78	0.28	0.33	0.85	Purifying selection	1.67
*CaANK*82-*CbANK*57	130.25	424.76	0.03	0.06	0.54	Purifying selection	0.30
*CbANK*54-*CbANK*57	151.41	499.59	0.02	0.02	0.73	Purifying selection	0.12
*CaANK*36-*CbANK*28	87.41	293.59	0.02	0.05	0.36	Purifying selection	0.24
*CbANK*84-*CbANK*28	88.09	292.91	0.02	0.02	1.05	Positive selection	0.12
*CbANK*35-*CbANK*33	69.45	245.55	0.14	0.24	0.60	Purifying selection	1.20
*CbANK*38-*CbANK*36	120.81	401.19	1.07	1.16	0.92	Purifying selection	5.87
*CaANK*77-*CaANK*38	80.09	306.91	0.03	0.08	0.38	Purifying selection	0.40
*CcANK*37-*CcANK*43	100.22	364.78	0.03	0.13	0.21	Purifying selection	0.66
*CcANK*60-*CcANK*48	102.73	371.27	0.07	0.14	0.51	Purifying selection	0.70
*CaANK*45-*CaANK*47	102.56	386.44	0.01	0.01	0.53	Purifying selection	0.05
*CbANK*31-*CaANK*47	102.70	386.31	0.01	0.02	0.26	Purifying selection	0.10
*CcANK*55-*CcANK*42	78.25	302.75	0.08	0.11	0.76	Purifying selection	0.55
*CaANK*50-*CcANK*40	97.15	361.85	0.08	0.21	0.38	Purifying selection	1.07
*CcANK*39-*CcANK*40	87.98	320.02	0.09	0.19	0.46	Purifying selection	0.97
*CbANK*56-*CbANK*53	228.17	797.83	0.04	0.09	0.44	Purifying selection	0.45
*CbANK*8-*CaANK*10	227.16	714.84	0.01	0.01	0.95	Purifying selection	0.07
*CbANK*7-*CbANK*5	287.24	921.76	0.01	0.01	0.93	Purifying selection	0.07
*CaANK*78-*CaANK*6	289.95	931.06	0.01	0.01	0.39	Purifying selection	0.07
*CbANK*11-*CbANK*6	139.63	481.37	0.03	0.04	0.77	Purifying selection	0.22
*CbANK*72-*CcANK*1	65.47	228.53	0.26	1.00	0.27	Purifying selection	5.02
*CcANK*80-*CcANK*1	64.38	226.62	0.27	1.03	0.26	Purifying selection	5.20

S-Sites, number of synonymous sites; N-Sites, number of non-synonymous sites; Ka, non-synonymous substitution rate; Ks, synonymous substitution; MYA, million years ago.

We further identified the rate of synonymous per synonymous site (Ks) and non-synonymous substitutions per non-synonymous site (Ka) values to explore the selective pressures on these paralog pairs and understand the expansion of this gene family in pepper. The value of the Ka/Ks ratio represents the type of selection pressure on the gene and evolutionary rate. Ka/Ks ~ 0 indicates that the selection is neutral, Ka/Ks < 1 indicates purifying selection and Ka/Ks > 1 indicates positive selection^37^. The Ka/Ks (ω) ratios for segmental duplications ranged from 0.15 to 1.05. In total, 22 of 23 paralog pairs were under purifying selection, with ω ratios < 1. These ratios suggest that the *ANK* gene family in these pepper species evolved by the removal of deleterious alleles. This type of selection preserves the long-term stability of genes over the course of evolution^37^. The ω ratio for *CbANK84*-*CbANK28* was >1, which indicates positive selection. Along with the selective pressures, we estimated the duplication time of *Capsicum* ANK paralog pairs by using a relative Ks measure as a proxy for time, and it spanned from 0.05 to 5.9 million years ago (MYA), with an average duplication time of ∼1.1 MYA.

### GO annotation of *Capsicum ANK* genes

GO analysis performed with Blast2Go suggested the putative participation of *ANK* genes in multiple biological processes, molecular functions, and cellular component (Fig. S6). For instance, most of the *ANK* genes are likely related to stress response, followed by signal transduction, and response to endogenous stimulus across all the *Capsicum* species. As well, in each of the three *Capsicum* species, we identified two *ANK* genes involved in transport, one in response to biotic stimulus, and one more in response to external stimulus. Analysis of the molecular functions predicted that the main roles of the *ANK* genes in all species were related to binding, transferase and kinase activities. Similarly, cellular component analysis revealed that 197 *ANK* genes were predicted in cell membrane, 9 were intracellular and 6 were in chloroplasts. These results provide useful information for future gene characterization studies in pepper.

### Capsaicinoid content in pepper

**Figure 4:**
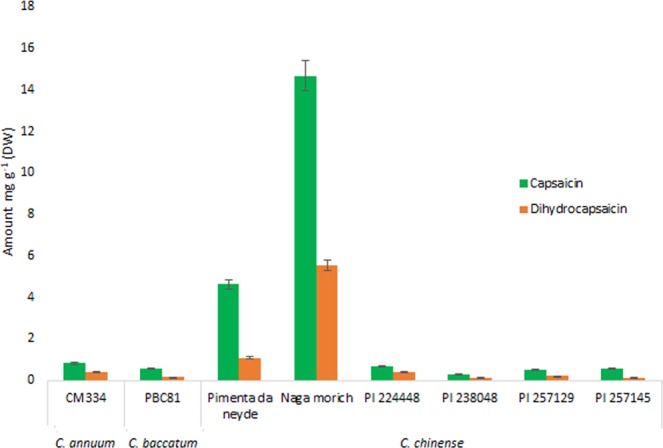
Capsaicin and dihydrocapsaicin content in pepper. Capsaicin and dihydrocapsaicin levels in pepper powder from dried green fruit (16 days post-anthesis [dpa]). Data are mean ± SD; n = 3.

To further explore the potential functions of *ANK* genes related with capsaicinoid content in pepper fruits, we firstly determined the capsaicin and dihydrocapsaicin amount in fruits at 16 dpa (days post-anthesis) from the three *Capsicum* species analyzed (Fig. 4). The highest content of capsaicin and dihydrocapsaicin was in *C. chinense* cv. Naga morich — 14.67 and 5.54 mg g^−1^ dry weight (DW) tissue, respectively — followed by *C. chinense* cv. Pimienta da neyde — 4.62 and 1.08 mg g^−1^ DW tissue, respectively. In contrast, for the remaining *C. chinense* varieties analyzed (PI 224448, PI 238048, PI 257129 and PI 257145), the content ranged from 0.28 to 0.68 mg g^−1^ DW for capsaicin and 0.13 to 0.4 mg g^−1^ DW for dihydrocapsaicin. Contrary to *C. chinense* fruits, for *C. annuum* cv. CM334, the content of capsaicin and dihydrocapsaicin was 0.823 and 0.393 mg g^−1^ DW tissue, respectively. The lowest content across all species was for *C. baccatum*, 0.55 and 0.15 mg g^−1^ for capsaicin and dihydrocapsaicin, respectively.

### *Ankyrin* genes associated with capsaicin in pepper

**Figure 5:**
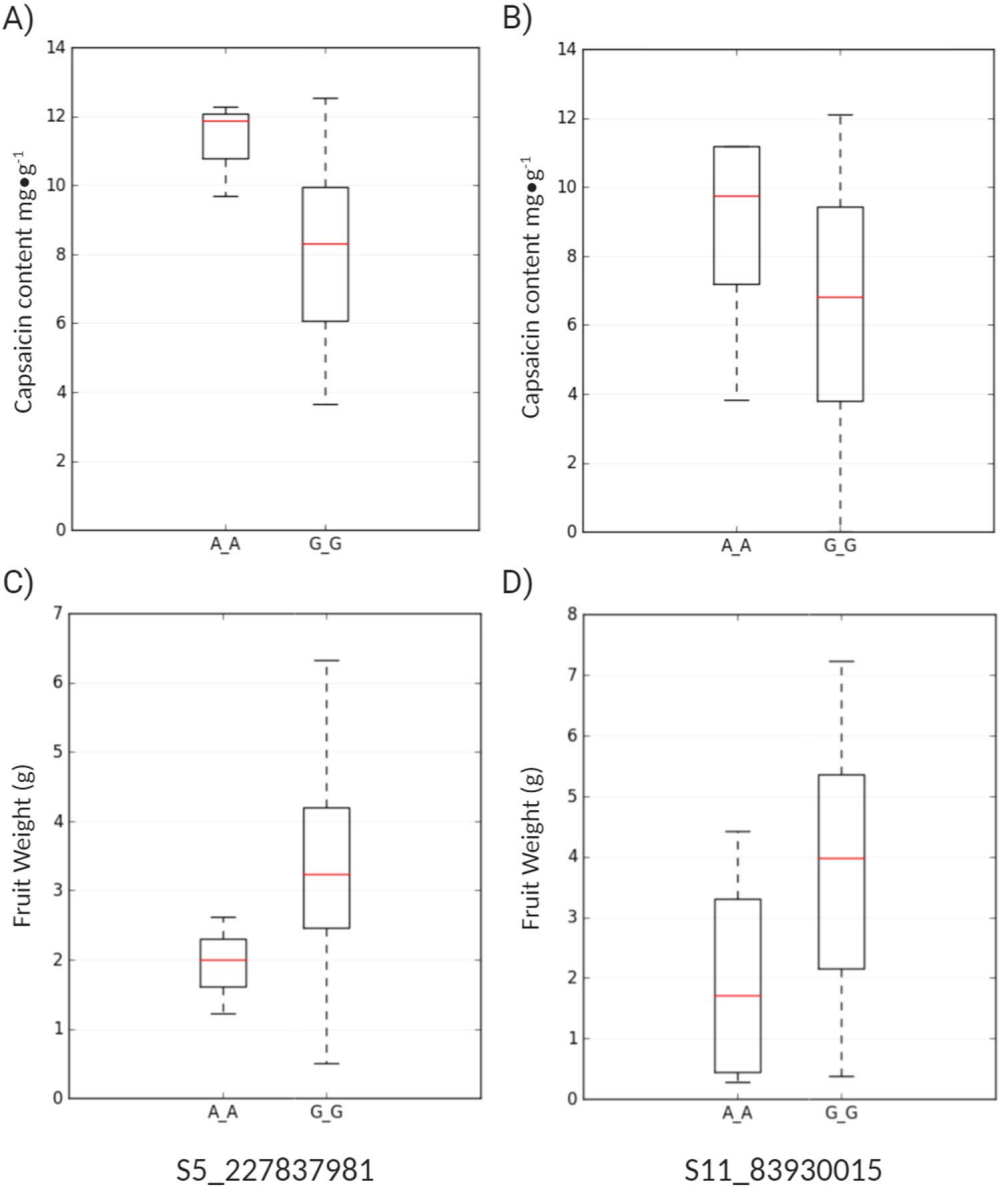
Allelic effect of significantly associated single nucleotide polymorphic (SNP) markers for capsaicin content in *C. annuum*. Boxplot shows the effect of SNP marker in locus (**A**) CA05g18080 on chromosome 05 and (**B**) CA1109160 on chromosome 11. Y-axis represents the values for capsaicin levels (mg·g^−1^) in pepper powder from dried green fruit (**A**,**B**) and total fruit weight (**C**,**D**).

Previous studies identified *ANK* genes that are involved in the biosynthesis of capsaicinoids and in the pungency modulation of pepper. For instance, in a genome-wide association study, Nimmakayala *et al*.^33^, reported two significant single nucleotide polymorphisms (SNPs) associated with capsaicin content, S5_227837931 and S11_83930015, were located in the locus CA05g18080, which codes for an ankyrin-like protein, and CA11g09160, which encodes a protein with acyltransferase activity, respectively. As shown in Fig. 5, the SNP positions S5_227837931 and S11_83930015, located in chromosome 5 and 11 respectively, have high allelic effect for capsaicin content and fruit weight; however, in both SNP positions, the non-pungent and high fruit weight cultivars contained a G allele, whereas the pungent and less fruit weight cultivars possessed A alleles. Likewise, Park *et al*.^34^, reported a major quantitative trait loci located on the locus CC.CCv1.2.scaffold31.147 of *C. chinense*, which encodes for an ankyrin-repeat–containing protein. This quantitative trait locus was suggested to play an important role in capsaicinoid biosynthesis of pungent pepper pericarps.

### Expression profile of *ANK* genes in *C. annuum* and *C. chinense*

**Figure 6:**
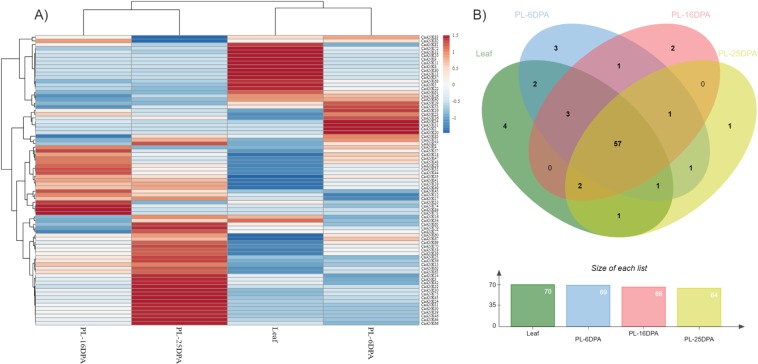
Expression patterns of *CaANK* genes in leaf and placenta tissue of *C. annuum* var CM344. (**A**) Heat map of expression profiles (in log2-based RPKM) from leaf and placenta tissue (6, 16, 25 days post-anthesis [dpa]). The expression levels are represented by the color: red, upregulated, and blue, downregulated. (**B**) Venn diagram analysis of the tissue expression of *CaANK* genes. PL, placenta.

We identified orthologs by a BLASTN strategy with two previously identified capsaicinoid markers (i.e., CA05g18080 and CA11g09160), which were obtained from the Sol Genomics Network database. The resulting orthologs were *CbANK41*, *CaANK44* and *CcANK61* for CA05g18080 and *CbANK73*, *CaANK68* and *CcANK81* for CA11g09160. To investigate the expression profile of individual *CaANK* genes across different tissues, including leaf and placenta (6, 16 and 25 dpa), we used publicly available RNA-seq data for *C. annuum* cv. CM334^28^. An expression heat map was used to visualize the *CaANK* tissue-specific expression patterns (Fig. 6). Overall, 81 out 85 genes were expressed in at least one of the tested tissues (Fig. 6A). However, only 57 genes were expressed in all assessed tissues and at various expression levels. Moreover, some genes exhibited unique expression profiles in a specific tissue. For instance, we found 4, 3, 2 and 1 tissue-specific *CaANKs* in leaf, 6-dpa, 16-dpa and 25-dpa placenta, respectively (Fig. 6B). *CaANK44* was highly expressed in placenta tissue at 16-dpa but moderately at 25-dpa and almost not expressed in leaf tissue (Fig. 6A). However, *CaANK68* showed a high expression pattern in leaf tissue versus placenta tissue at the three different stages.

We analyzed the gene expression of different ANK subfamilies. A member of the ANK-BTB subfamily (*CaANK32*) was highly expressed in leaf, whereas another member of the same subfamily (*CaANK55*) showed high expression in placenta tissue at 16-dpa. Likewise, *CaANK28* and *CaANK17*, members of the ANK-ACBP and ANK-GPCR subfamilies, respectively, showed similar expression patterns and were highly expressed in placenta at 16-dpa. Members of the ANK-ZnF subfamily (*CaANK3* and *CaANK*66) were highly expressed in placenta tissue at 25 dpa. *CaANK70*, which belongs to the ANK-BPA subfamily, was highly expressed in the same tissue and stage. Members of the ANK-U and ANK-O subfamilies were evenly expressed across all tissues and stages.

**Figure 7:**
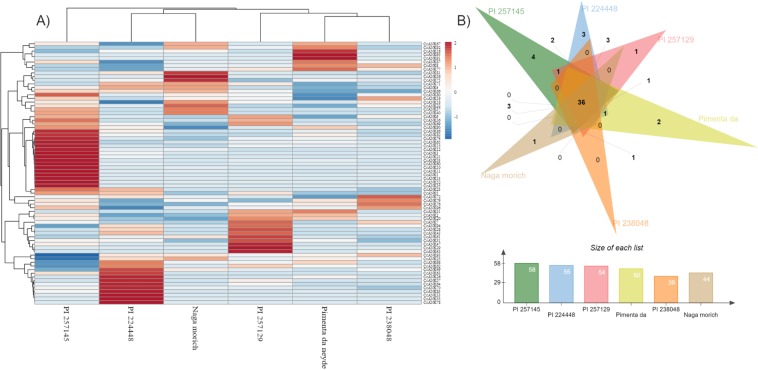
Expression patterns of CcANK genes in placenta tissue from six varieties of *C. chinense*. (**A**) Heat map of expression profiles (in log2-based RPKM) from placenta tissue at 16 dpa for six *C. chinense* cultivars. The expression levels are represented by the color: red, upregulated, and blue, downregulated. (**B**) Venn diagram analysis of the cultivar expression of *CcANK* genes. PL, placenta.

We analyzed the expression profile of *CcANKs* genes on the basis of their RPKM values from RNA-seq data of different *C. chinense* varieties, generating a hierarchical cluster and the expression profile of genes in placenta tissue at 16-dpa based on the log values of each gene (Fig. 7). Among 96 *CcANK* genes, 72 were expressed in at least one *C. chinense* cultivar (Fig. 7A) and 36 were expressed across all six cultivars (Fig. 7B). Four *CcANK* genes (*CcANK1*, *CcANK13*, *CcANK35*, *CcANK52*) were exclusively expressed in PI257145, whereas *CcANK33*, *CcANK55* and *CcANK78* were only found in PI224448. For PI257129, only *CcANK29* was uniquely expressed, whereas *CcANK95* and *CcANK91* were exclusively expressed in Pimenta da neyde. *CcANK61*, previously described as a major marker for capsaicin and dihydrocapsaicin content, was mostly expressed in PI257129 and with moderate expression in Naga morich, whereas *CcANK81* was highly expressed in this last variety. *CcANK65*, another candidate marker associated with capsaicin content in *C. chinense*, was highly expressed in PI257145 but with almost no expression in the other cultivars. In order of subfamilies, mainly ANK-U and ANK-O were unevenly expressed across all six cultivars, whereas the ANK-ZnF and ACBP subfamilies showed high expression in the Pimenta de neyde variety. Another member of the ANK-ZnF subfamily, *CcANK2*, was moderately expressed in PI257129. *CcANK23* showed moderate expression in PI224448 and PI257145. *CcANK10* and *CcANK80*, members of the ANK-GPCR and ANK-PK subfamilies, respectively, were highly expressed in PI257145.

**Figure 8:**
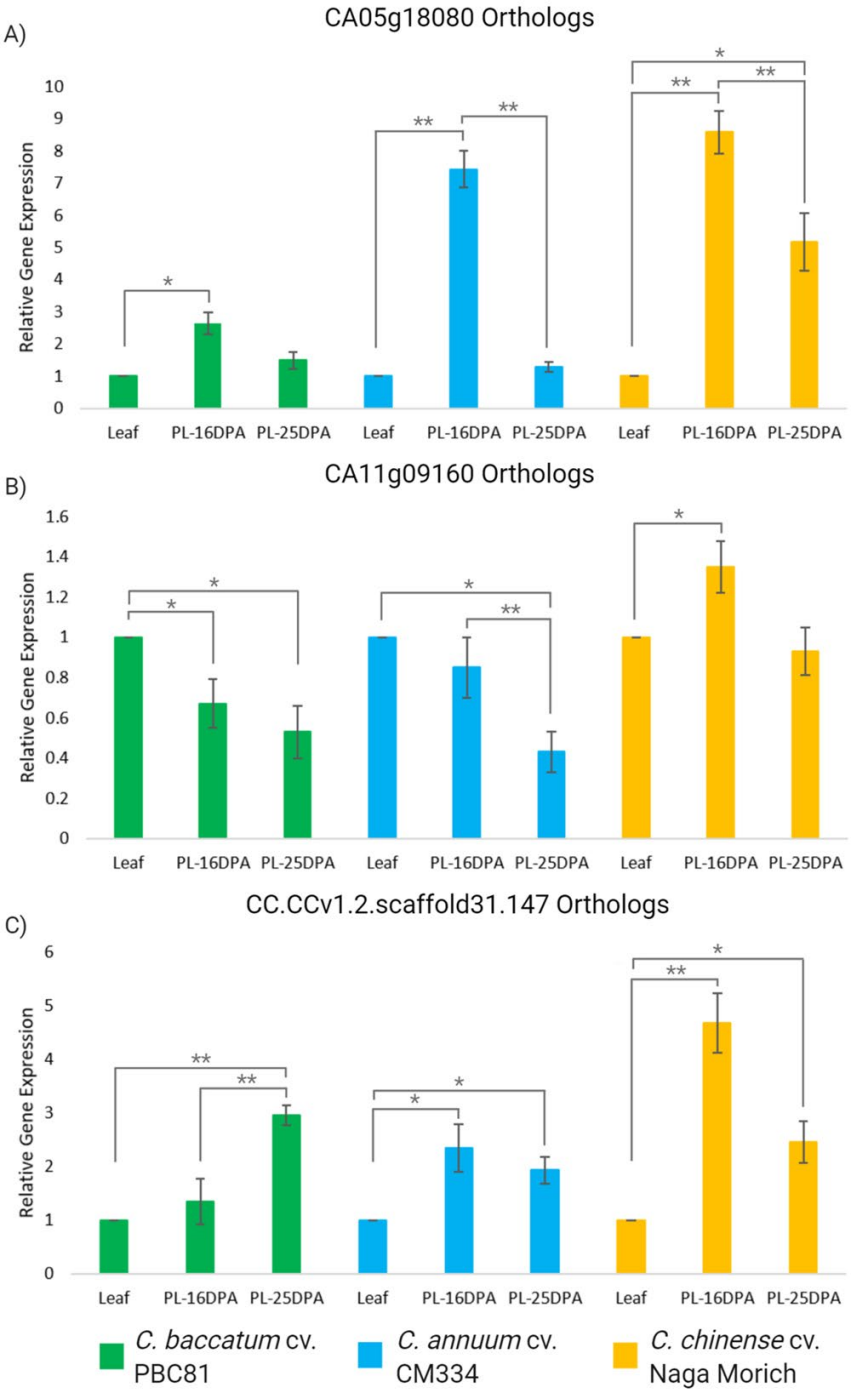
RT-qPCR analysis of mRNA expression of (**A**) CA05g18080, (**B**) CA11g09160 and (**C**) CC.CCv1.2.scaffold31.147 orthologs in leaf and placenta tissues at 16 and 25 dpa (days post-anthesis) across *Capsicum* species. Leaf was used as a calibrator for relative expression. Data are mean ± SD; n = 3, with significant differences as p ≤ 0.05 (*) and p ≤ 0.001 (**). PL, placenta.

Although the RNA-seq data provided relevant information about the expression pattern of *ANK* genes, there might be possible bias in the analysis. To confirm this information, we determined the preferential expression of CA05g18080, CA11g09160 and CC.CCv1.2.scaffold31.147 ortholog genes by using real-time PCR analysis of *ANK* genes in leaf and placenta tissue from the three *Capsicum* species at 16 and 25 dpa by using gene-specific primers (Fig. 8). The results showed a predominant expression of CA05g18080 orthologs in placenta at 16 dpa in *C. chinense*, followed by *C. annuum* at the same stage in placenta tissue with significant difference at p ≤ 0.001 compared to leaf and 25 dpa in both species. Similarly, the CA05g18080 ortholog had high expression in placenta tissue from *C. chinense* at 25 dpa. However, genes from *C*. *baccatum* showed the lowest expression levels, although the placental expression at 25 dpa was higher than in C. *annuum* at the same stage but not significant difference was found related to leaf and 16 dpa (Fig. 8A). In addition, the CA11g09160 ortholog from *C. chinense* showed high transcript abundance in placental tissues at 16 dpa, with lower expression at 25 dpa but these results were not significant. Contrary to *C. chinense*, the expression of this gene in *C. baccatum* and *C. annuum* was lowest in placenta tissues compared to leaf with significant difference at p ≤ 0.05 (Fig. 8B). Similarly, the orthologs of CC.CCv1.2.scaffold31.147 were significantly expressed in *C. chinense* at 16 dpa (p ≤ 0.001), followed by *C. annuum* (p ≤ 0.05) compared to leaf, while there was not significant difference in *C. baccatum* at the same stage. The expression in placenta tissue at 25 dpa was high in *C. baccatum* showing significant difference at p ≤ 0.001 in comparison to leaf and 16 dpa, nevertheless this expression was lower when comparing with *C. chinense* and *C. annuum* (Fig. 8C).

## Discussion

ANK proteins are known to play important roles in various developmental processes of different plant species such as *Arabidopsis*, rice, maize, soybean and tomato^18,23–25,27^. Although the role of *ANK* genes in plants was previously suggested, no systematic studies of the *ANK* gene family in *Capsicum* spp. have been performed; however, the recent availability of the pepper genome sequence has provided material support for the identification and characterization of varied gene families.

In this study, we identified 87 *ANK* genes in *C. baccatum*, 85 in *C. annuum* and 96 in *C*. *chinense*. All these genes were distributed among the 12 chromosomes at different densities across the three species. ANK repeat-containing proteins generally represent from 0.1% to 0.5% of the total proteins in most of the plants studied; however, this content can reach as much as 2% in plants such as *Ectocarpus siliculosus*^38^. In pepper, this percentage of the annotated protein-coding genes ranged from 0.24% in *C. baccatum* and *C. annuum* to 0.27% in *C. chinense* (Table 1). Thus, the number of *ANK* genes is lower in pepper than other plant species such as *Arabidopsis*, rice, and tomato^18,23,24^, which contain 0.41%, 0.49% and 0.38% of *ANK* genes, respectively. Given that the genome size of pepper is 3,500 Mbp^39^, nearly 26 times larger than the *Arabidopsis* genome (135 Mbp)^40^, about 8 times larger than the rice genome (430 Mbp)^41^, and almost 4 times the size of the tomato genome (950 Mbp)^42^, it is surprising than the number of *ANK* genes is much lower in pepper than in other plants. This paradox has also been observed in maize, with only 71 *ANK* genes reported in a genome size of 2,300 Mbp^25^.

In comparison to other gene families such as ABC transporters, the number of *ANK* genes in pepper is low^43^. However, this abundance is high as compared with other families such as the ARF^31^, DHN^30^, DIR^32^ and GRAS^44^ families, with 19, 7, 24 and 50 members, respectively. In general, most ANK proteins have 2 to 6 repeats, with a maximum of 34 repeats, which was reported in a *Giardia lamblia* protein^45^. In this study, we found that *C*. *chinense* contained proteins with 14 and 18 ANK repeats, whereas *C*. *annuum* proteins had 17 repeats (Fig. S1).

The predicted proteins were classified into 10 subfamilies on the basis of their domain compositions, and a combined phylogenetic tree was constructed with the aligned *Capsicum* and *Arabidopsis* ANK protein sequences. Most of the members within the same group shared one or more different domains in addition to the ANK domain. Gene structure analysis showed that pepper ANK subgroup members contained a similar organization in terms of the number and length of introns and exons. For instance, CA05g18080 orthologs in *C. baccatum* and *C. chinense* had two exons, whereas *C. annuum* had four exons. Similarly, CA11g09160 orthologs contained two exons in all three *Capsicum* species.

Although other plant species contain more *ANK* genes^23,24^ than do pepper species*, Capsicum* species contain more ANK proteins with an exclusively ANK domain (48–64%), classified in the ANK-U subfamily. The second largest group after the ANK-U subfamily was the ANK-TM subfamily. The orthologs of CA05g18080 and CA11g01960 were classified in the ANK-U subfamily, and the locus CC.CCv1.2.scaffold31.147 (*CcANK65*), homolog of At5g02620, was a member of the ANK-TM subfamily. Members of the ANK-TM family such as *AKT1*, which contains five ANK repeats and several transmembrane domains, play a key role in nitrogen fixation in root nodules of *Lotus japonicus*^46^ and in root K^+^ uptake^47,48^.

The *Capsicum ANK* genes included the ANK-BTB subfamily, which are known to be involved in plant morphogenesis and serve as a protein–protein interaction motif in several transcription factors^49,50^. The BLADE-ON-PETIOLE1 (BOP1) gene, which encodes for a BTB/POZ with an ANK repeat domain architecture, regulates the expression of the class I KNOX gene and modulates meristematic activity in leaves^16^. All *Capsicum* species analyzed had one gene belonging to the ANK-ACBP (Acyl-CoA-binding protein) subfamily and one more from the ANK-GPCR subfamily, which contains a GPCR-chapero-1. This last subfamily was previously reported in tomato and soybean, but we have no evidence demonstrating its presence in model plants such as *Arabidopsis*, rice or maize. We did not identify ANK proteins having the ring finger domain (ANK-RF subfamily) or tetratricopeptide repeat domain (ANK-TPR) in any of the three species, even though these domains have been identified at different densities in tomato, *Arabidopsis*, rice, maize and soybean^18,23–25,27^. Another identified subfamily was ANK-PK, with protein kinase activity. Previous studies described that the interaction between the receptor-like kinase complex and an ANK repeat domain can activate downstream defense signaling components, which suggests a role for ANK proteins in biotic stress^51^. For instance, Chinchilla *et al*.^52^, reported that the expression of an ANK protein kinase in alfalfa was induced by an osmotic effect.

Motif analysis with MEME revealed that the motifs *ACD6* and *ANK* were highly related. Earlier studies reported that the expression of *ACD6*, a protein containing a transmembrane region and a putative ankyrin repeat, was directly regulated by SA, which is highly required in response to plant pathogens and diseases^53,54^. Likewise, the gain of function of the *acd6-1 Arabidopsis* mutant was influenced by SA and light to activate an immune response against pathogens during infection^54^. These findings support plant ANK proteins as playing a crucial role in biotic stress response, mainly against pathogens and diseases. Nodzon *et al*.^55^, found that the expression of some *ANK* genes in plants can be affected by auxin, ABA and SA/jasmonic acid, which principally mediate the responses of plants to biotic and abiotic stress. The promoter elements found in *Capsicum ANK* genes were related to auxin, ABA and SA, and to GA, which has been reported to increase the growth and yield of *C. annuum* under greenhouse conditions^56^. Seong *et al*.^57^, demonstrated that the expression of *CaKR1* (Ankyrin Repeat-Containing Zinc Finger Protein) in *C. annuum* was strongly induced by SA and an ethylene regulator. One group of 11 *Cis*-elements was associated with binding protein site function, which supports that ANK proteins are evolutionarily conserved protein domains involved in mediating protein–protein interactions in different molecular process. Additionally, five *Cis*-elements were related to dehydration and water stress. Some ANK proteins may be involved in transpiration and adaptation to water deprivation stress, which agrees with the above results. Disruption of *AKT1* in *Arabidopsis* mutants increased stomatal closure during water deficit, thereby enhancing drought tolerance^58^.

The number of *ANK* genes is higher in *C. chinense* than other pepper species, so this species may be more resistant to drought stress while containing a higher amount of capsaicinoids^59,60^. Indeed, hot pepper cultivars with high capsaicinoid content are less sensitive to drought stress than are low and medium pungent cultivars^61^. Nevertheless, this feature cannot be generalized because many factors affect capsaicinoid content, including genotypic variation^62^. For instance, the different *C. chinense* varieties used in this study contained different capsaicinoid content (Fig. 4), which confirms that the pungency level is cultivar-dependent in part^63,64^.

Gene duplication events are important for the rapid expansion and evolution of gene families and are crucial for the origin of new gene functions^65^. Among the different types of duplication, segmental and tandem duplication are the most common involved in gene family expansion^66^. In our study, the average duplication time of syntenic *Capsicum* ANK paralogs pairs was ~1.1 MYA, which is close to the estimated lineage-divergence times of *C. baccatum* and a progenitor of the other two pepper species (~1.7 MYA). However, the duplication time between *C. annuum* and *C. chinense* was reported as 1.14 MYA^67^. We identified a total of 41 *Capsicum ANK* genes in 23 pairs of syntenic paralogs across all the species. The selection pressure type was measured according to the ratio of non-synonymous to synonymous substitutions ω (=*Ka/Ks*). The *Ka/Ks* ratios of 22 paralog pairs were <1, so these paralogs were under purifying selection pressures. Furthermore, the ω ratio of one paralog pair was >1, representing positive selection and fast evolutionary rate. These findings are similar to those for other gene families such as BURP in Medicago and ACD in tomato, which contain a few or even no paralog pairs undergoing positive selection^68,69^.

Most of the *Capsicum* ANK products were predicted to be membrane, intracellular and chloroplast proteins. ANK repeats have been found in proteins with different functions, including mitochondrial proteins, cell cycle regulation, cytoskeleton interactions, signal transduction, disease resistance and stress responses^1,70^. We found that *Capsicum ANK* genes are mainly involved in response to stress and endogenous stimulus and are involved in signal transduction in all *Capsicum* species. The response to endogenous stimulus as a biological process supports the participation of *ANK* genes in stress tolerance, as was shown in other species. For example, the ankyrin repeat-containing XA21 binding protein 3 (*XB3*), which forms a protein complex with receptor-like kinase (*XA21*), confers resistance to bacterial blight caused by *Xanthomonas oryzae* in rice^51^. Similarly, the *OsPIANK1* gene positively regulates rice basal defense against blast fungus disease^22^. The ANK repeat–receptor-like protein complex mediates the plant immune response, but *AKR2* plays a role in plant reactive oxygen species scavenging and metabolism^10^, and the OXIDATIVE STRESS 2 protein may be an activator in a stress response pathway^71^.

*CaANK* genes exhibited stage-specific expression, whereas *CcANK* genes showed a genotype-specific expression pattern, which suggests that the expression profile of these genes differs across *Capsicum* species. Furthermore, different patterns in the expression between orthologs of the Capsaicinoid markers previously mentioned demonstrated that the expression of *Capsicum ANK* genes was extensive during different development stages and may be species-specific for each of the *ANK* genes in *Capsicum* species. Overall, the identification of the putative *Capsicum ANK* genes, classification of these genes, construction of a phylogenetic tree, analysis of gene structure and more detailed knowledge of the expression profile of the *Capsicum ANK* genes may provide clues regarding the function of this gene family. Our findings provide new insights into the *Capsicum ANK* gene family, which helps improve our understanding of the possible role of these genes in aspects such as plant and fruit development, response against stress, and capsaicinoid content in pepper fruits.

## Materials and Methods

### Plant materials

*C. baccatum* cv. PBC81, *C. annuum* cv. CM334 and six varieties of *C. chinense* (i.e., Pimenta da neyde, Naga morich, PI 224448, PI 238048, PI 257129 and PI 257145) were grown in triplicate samples in an experimental field at West Virginia State University, adapting a row-to-plant spacing of 100 × 30 cm. Leaf and fruits at 6, 16 and 25 days post-anthesis (dpa) were collected from all cultivars and stored at −80 °C. Quantitative analysis of capsaicin and dihydrocapsaicin content in green pepper fruits (16 dpa) involved using the 1200 series HPLC system (Agilent Technologies, Santa Clara, CA) as described^72^.

### Identification of ANK genes in pepper

To identify all candidate members of the *ANK* gene family in pepper genomes, we downloaded the previously reported ANK protein sequences from *Arabidopsis*^23^ from the TAIR database (https://www.Arabidopsis.org/) and used them as a query in a local BLASTP with E-value cut-off of 1e-3 against the proteomes of the three *Capsicum* species downloaded from the Pepper Genome Platform (PGP) (http://passport.pepper.snu.ac.kr/?t=PGENOME)^28^. The typical ANK repeat domains PF00023 and SM00248 were searched across all the output genes by using the Pfam web server (http://Pfam.sanger.ac.uk/)^73^ and SMART database (http://smart.embl-heidelberg.de/smart/set_mode.cgi?NORMAL=1)^74^ respectively, with default cut-off parameters. Genes with *E*-value > 1E-05 and redundant genes were deleted. Analysis of the domain structure for all the peptide sequences of *ANK* genes selected involved using the latest Hidden Markov Model (HMM) with the HMMER3.0 software^75^. Protein size, MW and theoretical pI of each *ANK* gene were predicted by using the proteome database and sequence analysis tools on the ExPASy Proteomics Server (http://expasy.org/)^76^.

### MEME motif analysis

Conserved motifs of all ANK proteins from the three *Capsicum* species were identified by using the motif investigation software Multiple Em for Motif Elicitation (MEME) (http://meme-suite.org/tools/meme)^77^. The analysis was performed with maximum number of motifs 10 and optimum width of motif from 6 to 50. To identify motif function, discovered MEME motifs were searched in the ExPASy-PROSITE database by using the ScanProsite tool (https://prosite.expasy.org/scanprosite/)^78^. Prediction of the tertiary structure and homologs of ANK domain-containing proteins associated with capsaicin content in pepper for the three *Capsicum* species involved using the online server Phyre2 (Protein Homology/Analog Y Recognition Engine; http://www.sbg.bio.ic. ac.uk/phyre2) under the “intensive” mode as described by Kelley *et al*.^79^.

### Chromosomal location and gene structures of ANK genes

The chromosomal position of individual *ANK* genes was extracted from the PGP and the genes were physically mapped on each of the 12 chromosomes by using MapInspect. Structural analysis of ANK genes was generated by using the Gene Structure Display Server (GSDS: http://gsds.cbi.pku.edu.cn/)^80^. For *Cis*-element analysis, all *ANK* gene promoter sequences (1,500 bp upstream of the initiation codon “ATG”) were extracted from the pepper genomes. Then, the *cis*-regulatory elements of promoters for each gene were identified by using PLACE: A database of plant *cis*-acting regulatory DNA elements (http://www.dna.affrc.go.jp/PLACE/)^81^.

### Sequence alignment and phylogenetic analysis

The full-length ANK protein sequences from *Arabidopsis* and *Capsicum* species were aligned by using ClustalW as described^82^. The alignment file was then used to construct a phylogenetic tree based on the maximum-likelihood method of the MEGAX (Molecular Evolutionary Genetics Analysis) software^83^. The phylogenetic tree of the ANK proteins was displayed by using the interactive Tree Of Life platform (iTOL; http://itol.embl.de/index.shtml) after bootstrap analysis with 1000 replicates^84^.

### Gene synteny analysis of ANK proteins

The syntenic ANK paralog pairs across the *Capsicum* species were identified by searching gene duplication with the following criteria: (1) genes with >70% coverage of the alignment length; (2) genes with >70% identity in the aligned region; and (3) a minimum of two duplication events considered for strongly connected genes^85^. For each paralog pair, the non-synonymous substitution rate (Ka), synonymous substitution rate (Ks) and ω (=Ka/Ks) of paralog pairs were estimated by using KaKs_Calculator 2.0^86^. The duplication date of paralog pairs was estimated by the formula T = Ks/2λ, assuming a clock-like rate (λ) of 6.96 synonymous substitutions per 10^−9^ years^87^. The syntenic plot was generated by using the BioCircos package in R.

### cDNA library construction and RNA-seq of *C. chinense* green fruits

Green fruits (16 dpa) from six different cultivars of *C. chinense* were used for whole-transcriptome sequencing. Total RNA was isolated from the pooled tissues of three biological replicates for each cultivar by using the plant RNA mini spin kit (Macherey-Nagel). Total mRNA was isolated, fragmented, and reverse-transcribed into cDNA. Double-stranded cDNA was then purified by using 1.8x Agencourt AMP XP beads. Sequencing libraries were constructed by using the NEBNext Ultra II RNA Library Prep Kit according to the manufacturer’s protocol. To confirm accuracy before sequencing, the insert size and integrity of the libraries were analyzed with an Agilent 2100 Bioanalyzer (Invitrogen), and the Qubit 4 Fluorometer (Invitrogen) was used for library quantification. The RNA sequencing library from each sample was sequenced in the Illumina NextSeq. 500 platform to produce paired-end reads.

### Sequencing analysis and functional annotation

High-quality reads were obtained by removing the adapter sequence with Cutadapt and low-quality reads (Phred score QV < 30) with the Sickle program^88,89^. All cleaned reads were mapped to the *C. chinense* reference genome version 1.2 by using the mem algorithm of the BWA tool to generate a SAM alignment. The read count table for genes from *C. chinense* was created for all samples by using the SAM alignment and HTSeq R package^90,91^. The gene expression based on the read counts were normalized by calculating the reads per kilobase of transcript per million mapped reads (RPKM) values. The RPKM values for each gene were calculated by using the read count table, total number of reads, and gene length (kb). Found genes were functionally annotated to analyze their gene ontology (GO) by using the Blast2GO application (http://www.blast2go.com)^92^.

### Expression pattern of ANK genes in *C. annuum* and *C. chinense* and qRT-PCR validation

RNA sequencing (RNA-seq) gene expression data of leaf and placenta tissues (6, 16, 25 dpa) from *C. annuum* cv. CM334 were retrieved from the RNA-seq data published by Kim *et al*.^28^. The RPKM expression values were used to generate a heatmap for *ANK* genes from *C. annuum* and *C. chinense* by using the ClustVis web tool (https://biit.cs.ut.ee/clustvis/)^93^. RNA-seq data were further validated by qRT-PCR analysis of samples from different tissues (i.e., leaf, and placenta tissues at 6, 16 and 25 dpa). Synthesis of cDNA involved 1 μg total RNA with oligo dT primers and SuperScript IV reverse transcriptase. The resulting cDNA was subjected to RT-qPCR analysis with a total volume of 20 μL containing 1 μL cDNA template, 2 μL forward and reverse primers (10 μM), 10 μL SYBR Green PCR Master (ROX) (Roche, Shanghai) and 7 μL sterile distilled water on a StepOne Plus Real-Time PCR System. The two step RT-qPCR program began at 95 °C for 10 min, followed by 40 cycles of 95 °C for 15 s and 60 °C for 1 min. The reactions were performed in three biological replicates with three technical replications to compute the average Ct values. The relative expression of specific genes was quantified with the 2^−ΔΔCt^ calculation method^94^. Relative gene expression of target genes was normalized to that of the endogenous control β-tubulin^95^ and leaf tissue as a calibrator. Student’s t-test was used to identify statistical significance between samples.

## Supplementary information

Supplementary Figures.

Dataset 1.

## Data Availability

The raw Illumina mRNA-seq reads generated and/or analyzed during the current study are available in the Sequence Read Archive repository (NCBI-SRA) under the following accession numbers PRJNA526219 (https://www.ncbi.nlm.nih.gov/bioproject/PRJNA526219) and PRJNA562491 (https://www.ncbi.nlm.nih.gov/bioproject/?term=PRJNA562491).
