# Supplementary material for: The ankyrin repeat gene family in Capsicum spp: Genome-wide survey, characterization and gene expression profile
Source: Sci Rep. 2020 Mar 4;10:4044. doi: 10.1038/s41598-020-61057-4 (PMC7055287; doi:10.1038/s41598-020-61057-4)
Supplement: Supplementary file 1 — Supplementary Figures. [file 41598_2020_61057_MOESM1_ESM.pdf]

The ankyrin repeat gene family in *Capsicum* spp: Genome-wide survey, characterization and gene expression profile

Carlos Lopez-Ortiz[1,¶], Yadira Peña-Garcia[1,¶], Purushothaman Natarajan[1,2], Menuka Bhandari[1], Venkata Abburi[1], Sudip Dutta[1,3], Lav Yadav[1], John Stommel[4], Padma Nimmakayala[1,*] and Umesh K. Reddy[1,*]

[1]Department of Biology, Gus R. Douglass Institute, West Virginia State University, Institute, West Virginia, United States of America.

[2]Department of Genetic Engineering, School of Bioengineering, SRM Institute of Science and Technology, Kattankulathur, 603203, India.

[3]ICAR RC NEH Region, Mizoram Centre, Kolasib, Mizoram, India.

[4]Genetic Improvement of Fruits and Vegetables Laboratory (USDA, ARS), Beltsville, MD 20705, USA.

[*]Corresponding author:

Umesh K. Reddy ureddy@wvstateu.edu; Phone: +1304 7663066

Padma Nimmakayala padma@wvstateu.edu; Phone: +1304 7663258

[¶]These authors contributed equally to this work.

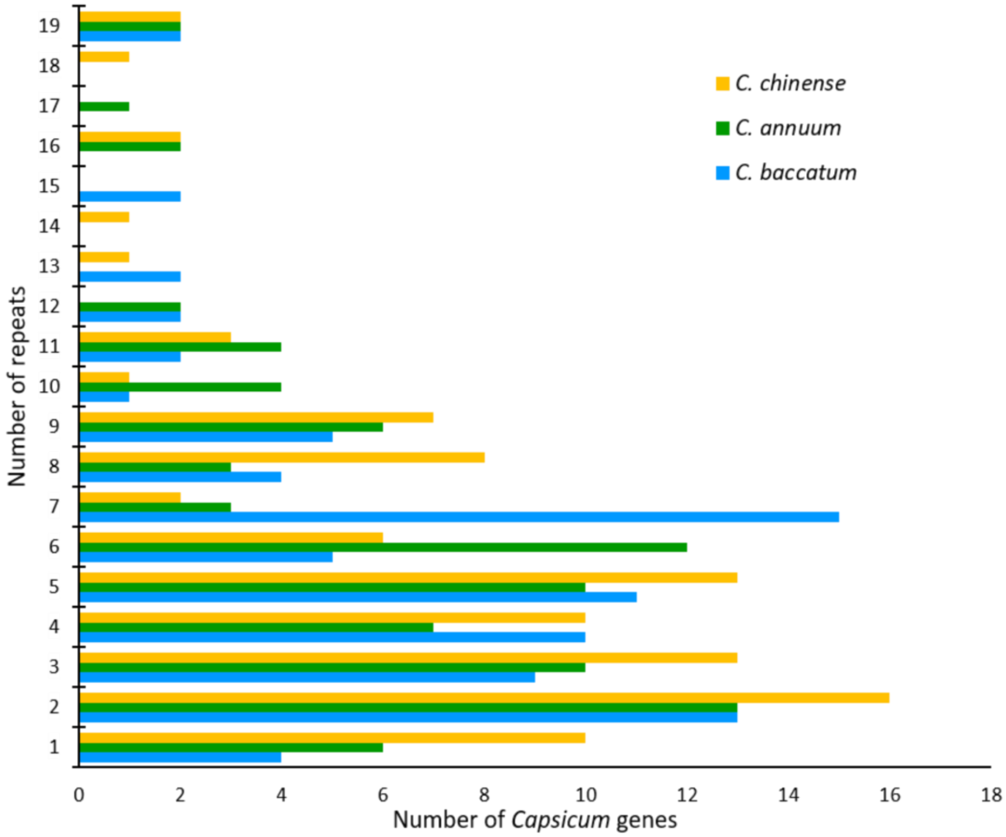

Fig. S1 Graphical representation of the number of predicted ankyrin (ANK) repeats per protein. The vertical axis represents different numbers of ANK repeats and the horizontal axis the frequency of the proteins corresponding to different number of repeats.

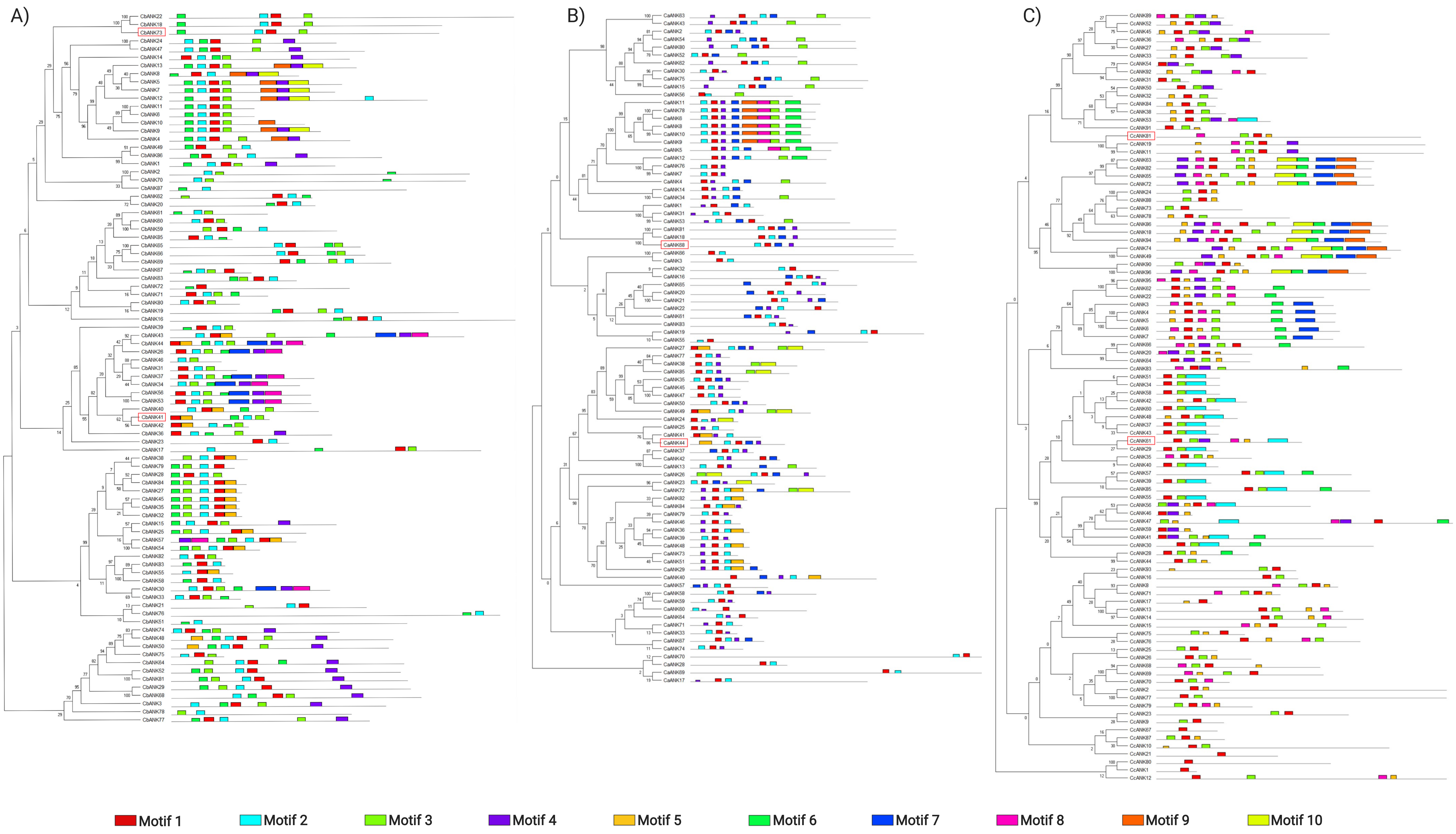

Fig. S2 Conserved motifs of *Capsicum* ANK genes according to evolutionary relationship. A) *C. baccatum*, B) *C. annuum* and C) *C. chinense*. The conserved motifs were elucidated by using MEME with complete protein sequences. Different motifs are represented by different colors, numbered 1–10 at the bottom. The black lines represent the non-conserved sequences.

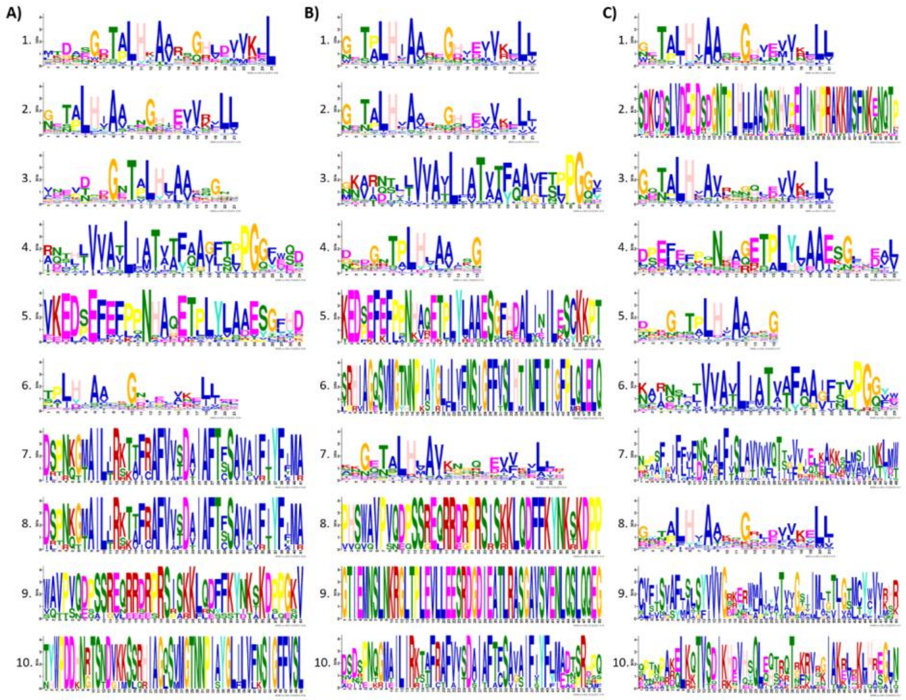

Fig. S3 Conserved motifs of ANK proteins in *Capsicum* species. (A) *C. baccatum*, (B) *C. annuum* and (C) *C. chinense*. The overall height of the stack indicates the degree of sequence conservation. The height of residues suggests the relative frequency of each residue at that position.

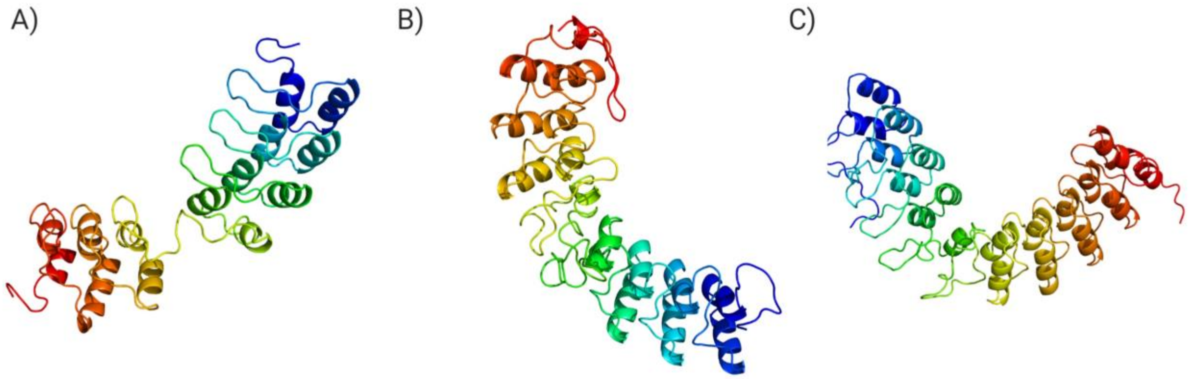

Fig. S4 Predicated structures of ANK proteins. The structure of ortholog proteins of CA05g18080 A), CbANK41 B), CaANK44 and C) CcANK61 with .90% confidence level are shown.

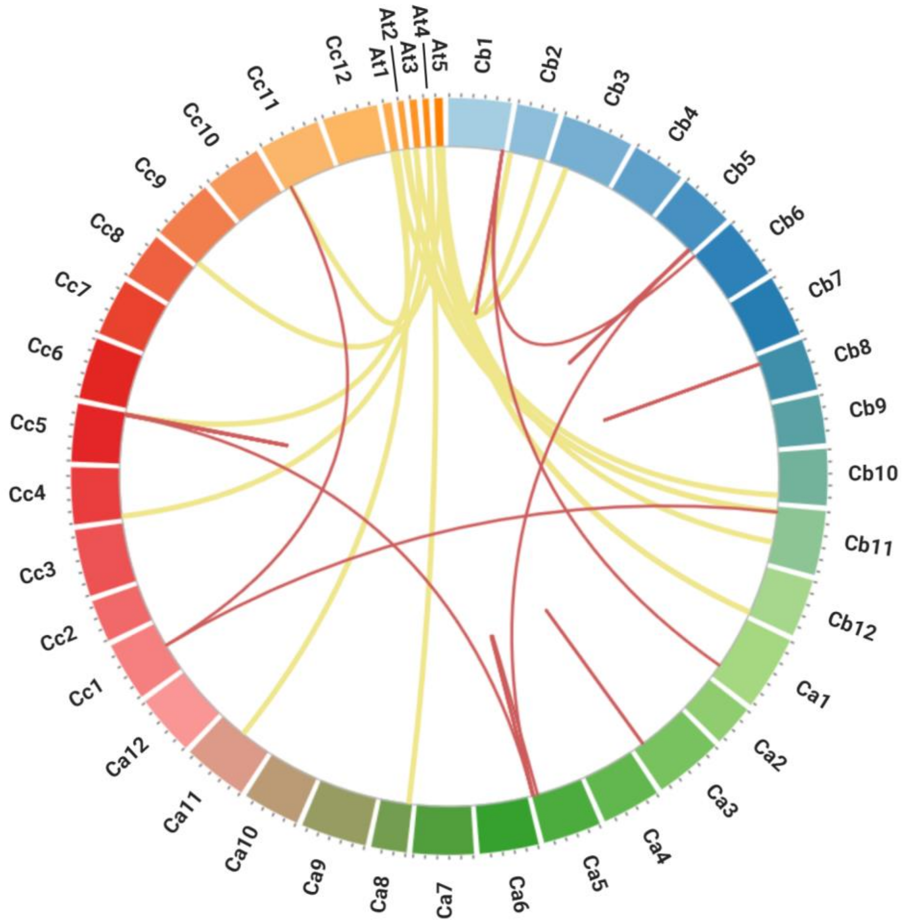

Fig. S5 Syntenic relationships among ANK genes of *C. baccatum* (Cb), *C. annuum* (Ca), *C. chinense* (Cc) and *Arabidopsis thaliana* (At). Red and yellow lines connecting two chromosomal positions indicate syntenic regions between all pepper species and pepper and *Arabidopsis* chromosomes, respectively.

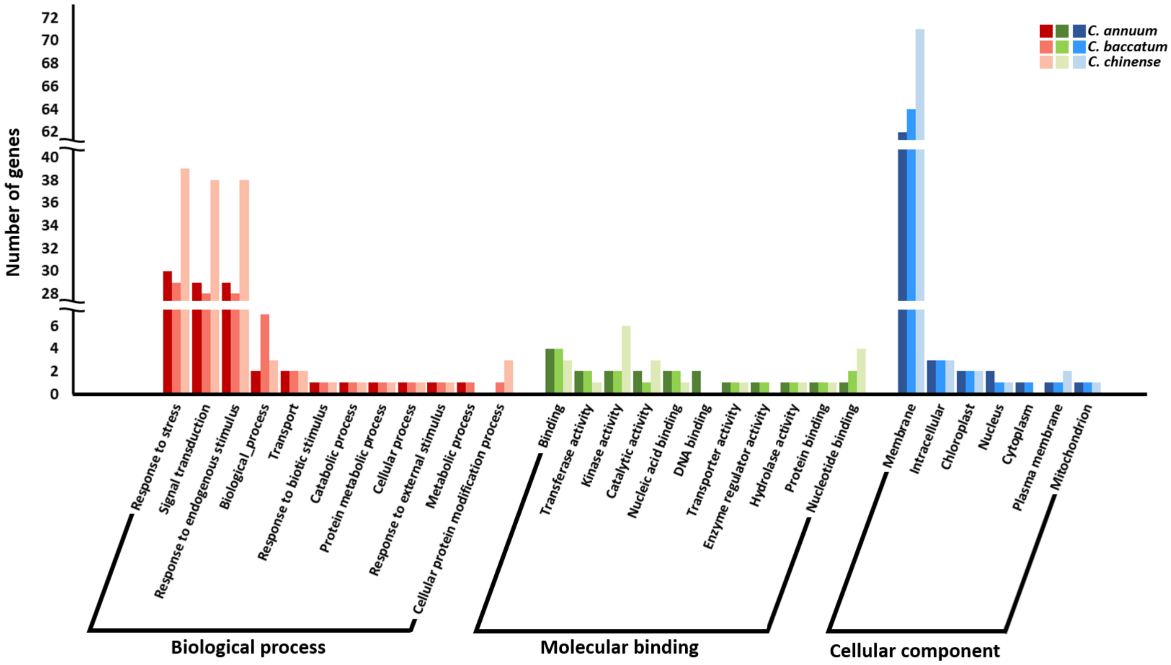

Fig. S6 Detailed Gene Ontology analysis for *Capsicum* species. Biological process, cellular component, and molecular function were identified by using the Blast2GO program.
